# Impact of external medium conductivity on cell membrane electropermeabilization by microsecond and nanosecond electric pulses

**DOI:** 10.1038/srep19957

**Published:** 2016-02-01

**Authors:** Aude Silve, Isabelle Leray, Clair Poignard, Lluis M. Mir

**Affiliations:** 1 Institute for Pulsed Power and Microwave Technology, Karlsruhe Institute of Technology, Germany; 2 Laboratory of Vectorology and Anticancer Therapies, UMR 8203, CNRS, Univ.Paris-Sud, Université Paris-Saclay, Gustave Roussy, PR2, Villejuif, 94805, France; 3 Inria Bordeaux, CNRS UMR 5251 & Université de Bordeaux, France

## Abstract

The impact of external medium conductivity on the efficiency of the reversible permeabilisation caused by pulsed electric fields was investigated. Pulses of 12 ns, 102 ns or 100 μs were investigated. Whenever permeabilisation could be detected after the delivery of one single pulse, media of lower conductivity induced more efficient reversible permeabilisation and thus independently of the medium composition. Effect of medium conductivity can however be hidden by some saturation effects, for example when pulses are cumulated (use of trains of 8 pulses) or when the detection method is not sensitive enough. This explains the contradicting results that can be found in the literature. The new data are complementary to those of one of our previous study in which an opposite effect of the conductivity was highlighted. It stresses that the conductivity of the medium influences the reversible permeabilization by several ways. Moreover, these results clearly indicate that electropermeabilisation does not linearly depend on the energy delivered to the cells.

Electropermeabilisation, also called electroporation, is one of the well documented effects of pulsed electric fields on plasma membrane^1^,^2^,^3^. It consists in applying electric pulses on cells in order to make the plasma membrane transiently or permanently permeable to a large variety of usually nonpermeant molecules.

Until the end of the 20^th^ century, conventional electropermeabilisation was obtained with pulses of a few microseconds or a few milliseconds (in this paper respectively referred to as ‘micropulse’, or ‘millipulse’). Several applications using such pulses have been successfully developed like electrochemotherapy^4^,^5^, electrogenetransfer^6^ or many applications in the food industry^7^.

More recently light was focused on the effect of much shorter pulses in the nanosecond range. Such pulses are referred to as nanopulses, nanosecond pulses or nanosecond pulsed electric field (nsPEF). Permeabilisation of the external cellular membrane induced by such pulses of a few nanoseconds or tens of nanoseconds has been reported several times using different permeabilisation markers like propidium^8^, thallium^9^, yo-pro^10^, calcium influx^11^ or bleomycin^12^. Whether nanopulses, micropulses and millipulses act on the membrane in a similar way is still an open question. To help clarifying this issue, we focused on the influence of extra-cellular medium on permeabilisation by both microsecond and nanosecond pulses.

Several groups have studied the influence of medium conductivity on membrane permeabilization by microsecond pulses^13^,^14^,^15^,^16^,^17^,^18^,^19^ (for a review see^20^). Some studies conclude that a decrease in extracellular medium conductivity slightly decreases the impact of the electric pulses^13^,^14^,^15^,^16^. According to these papers, such an influence is however very weak and can be observed only for very low values of conductivity, typically less than 0.01 S/m. Other studies, mainly from the group of Ulrich Zimmermann concluded on the contrary that an increase of extracellular medium conductivity tends to decrease the efficiency of permeabilisation^17^,^18^,^19^. The methods used to explore the influence of medium conductivity vary depending on the study. It can be cell death induced by the exposure to pulses^14^,^16^, release or uptake of molecules^13^,^16^,^17^,^18^ or more indirect effects like the expression of a transfected gene^15^. Two studies focused specifically on reversible membrane permeabilization and they either concluded that conductivity does not impact the efficiency of the reversible permeabilization^14^ or that lower conductivities are more efficient^17^.

The impact of extracellular conductivity on the efficiency of shorter pulses (in the nanosecond range) has been less intensively studied so far. A possible influence was suggested in a theoretical study where the authors considered that permeabilization by nanosecond pulses should be scaled with the charge density^21^. This hypothesis led to the conclusion that permeabilization should be more efficient in high conductivity media. Additionally, two experimental studies have already been published. The first one measured uptake of propidium iodide after delivery of a single pulse of 16 MV/m and with a duration between 11 ns and 95 ns^8^. It was concluded that uptake of PI was more efficient for the low extracellular conductivities, at least on the studied range −0.1 to 0.5 S/m. The other study was published recently by our group and indicated an opposite tendency: reversible permeabilization induced by 12-ns pulses of moderate magnitude 3.2 MV/m on DC3-F cells was detected in a medium with an external conductivity of 1.5 S/m and not when the conductivity was lowered to 0.1 S/m^22^. We hypothesized that the longer charging time of the cell’s membrane in a low conductivity medium did not allow sufficient membrane charging in order to trigger permeabilization, as discussed in detail in reference^22^.

The experiments reported here were designed to get a more global understanding of the impact of extracellular conductivity on electropermeabilization efficacy. In contrast to our previous study, here we restricted the experimental conditions to more intense treatment parameters, where permeabilisation could be detected after one pulse or very few pulses. Both very short pulses of 12 ns and 102 ns as well as more traditional long pulses of 100 μs were used. The study was focused on reversible electropermeabilization. Cell death due to bleomycin entry was used as diagnostic approach. Indeed, experiments in which the sole pulse-induced cell death is used as a diagnostic indicator are very difficult to analyze since cell death can occur through many different pathways and, more important for this study, can be very sensitive to the composition of the external medium^23^,^24^. Reversible electropermeabilization, in this study, was thus consistently detected by the uptake of the normally impermeant cytotoxic drug bleomycin. Under these conditions, and provided that pulse alone did not induce mortality, cell death occurred exclusively as a consequence of bleomycin penetration, representing a highly sensitive indicator for reversible electropermeabilisation of the cellular membrane.

In the following section we will present results on the degree of reversible membrane permeabilization as a function of the conductivity and the composition of the external medium, over a wide range of treatment parameters.

## Results

### Impact of conductivity for 100 μs pulses

**Figure 1:**
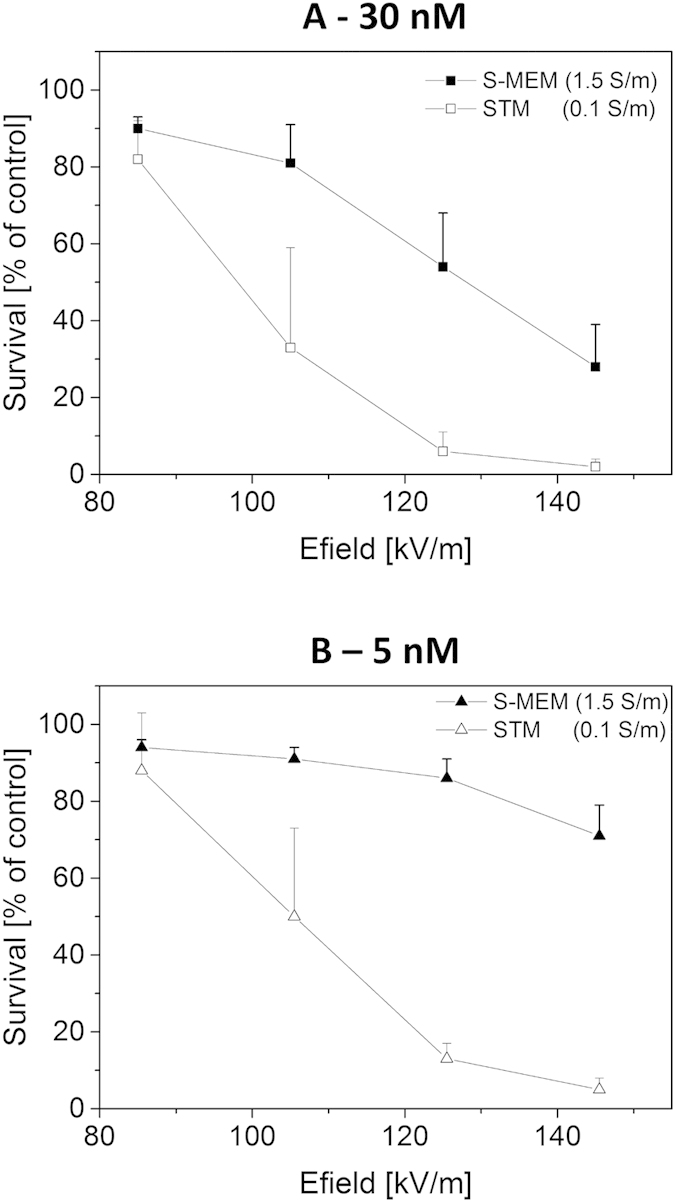
Cell survival as a function of pulse magnitude. Cloning efficiency tests were performed after exposure to one single micropulse of 100 μs in S-MEM or STM media containing either 30 nM (**A**) or 5 nM (**B**) bleomycin. Results are represented as mean values + SD (standard deviation) of three to five independent experiments. Cell survival is normalized with respect to the control submitted to the bleomycin alone. Note that dead cells are the ones reversibly permeabilized since pulse treatment alone (i.e. without bleomycin) did not induce any cell death (data not shown).

The first experiments were performed in S-MEM (conductivity 1.5 S/m) and in STM (0.1 S/m) with a single pulse of 100 μs. The external electric field was varied between 85 kV/m and 145 kV/m. Under these conditions, no cell death induced by the electric pulses alone, i.e. in the absence of bleomycin, was observed (data not shown). Consequently, in the presence of bleomycin, the fraction of cells that died corresponds to the fraction of cells reversibly permeabilized by the electric pulses. Our experiments were performed with a bleomycin concentration of either 30 nM (Fig. 1A) or 5 nM (Fig. 1B). One can see that for a given pulse, cell survival is always higher for the lowest bleomycin concentration. This corresponds to the observation that poorly electropermeabilized cells cannot incorporate the lethal dose of bleomycin molecules if the external bleomycin concentration is too low^12^. Indeed, It is known that the amount of bleomycin penetrating a permeabilized cell is proportional to the external concentration of bleomycin (at least for concentrations up to 100 μM^25^). For both bleomycin concentrations, survival rates were significantly lower in the STM medium indicating that electropermeabilization induced by one single pulse of 100 μs was more intense in the low conductivity medium. The influence of the extracellular medium was even more visible when the bleomycin concentration was 5 nM.

**Figure 2:**
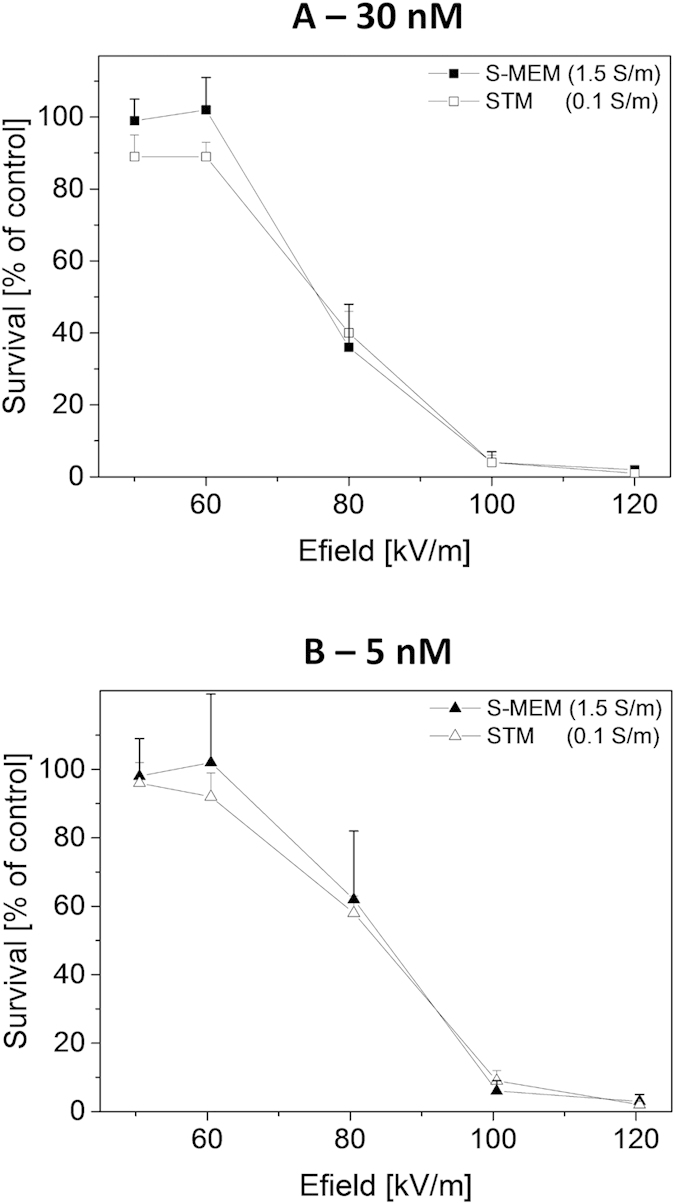
Cell survival as a function of pulse magnitude. Cloning efficiency tests were performed after exposure to a train of 8 micropulses of 100 μs applied with a repetition rate of 1 Hz. External medium was either S-MEM or STM containing either 30 nM bleomycin (**A**) or 5 nM bleomycin (**B**). Results are represented as mean values + SD of three independent experiments. Cell survival is normalized with respect to the control submitted to the bleomycin alone. Dead cells are the ones reversibly permeabilized since pulse treatment alone (i.e. without bleomycin) did not induce any cell death (data not shown).

At first view, this influence of the extracellular medium conductivity on cell reversible permeabilization seems to contradict a previous study of Pucihar and collegues^14^ performed with the same cells, the same media and also the same detection method (i.e. survival to the combination of electric pulses and bleomycin). In this previous study however, a different pulse protocol was used, consisting in a train of eight 100 μs-pulses applied with a repetition rate of 1 Hz. With such a pulse protocol, the authors observed no influence of the extracellular medium on reversible permeabilization. Experiments were repeated following exactly the same protocol. Results are displayed on Fig. 2. Again, the field intensities that were chosen did not induce any cell death in the absence of bleomycin (data not shown). However, reversible membrane permeabilization did occur as indicated by increased cell mortality when cells were challenged with the same pulses in the presence of bleomycin.

For both bleomycin concentrations tested, 30 nM and 5 nM, no difference in the efficiency of the reversible permeabilization between the two extracellular media was observed, confirming previous results of Pucihar and colleagues. Apparently, the influence of the conductivity of the external medium cannot be detected under these specific experimental conditions i.e. when several pulses are applied.

**Figure 3:**
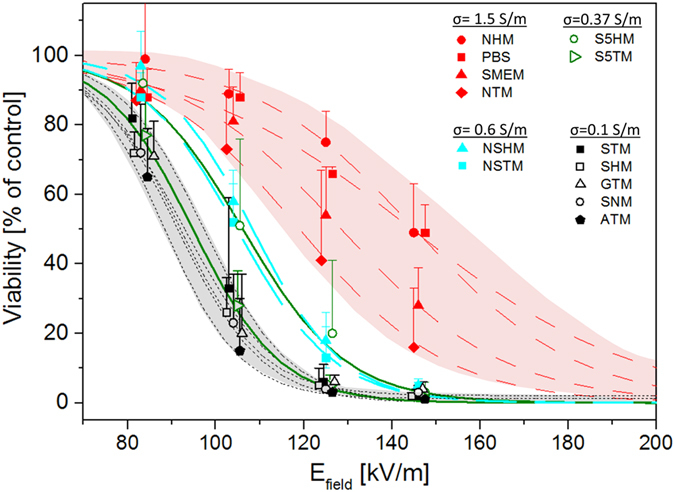
Cell survival as a function of pulse magnitude for different extracellular media. Cloning efficiency tests were performed after exposure to one single micropulse of 100 μs. External medium always contained 30 nM bleomycin. Results are represented as mean values + SD of three to five independent experiments. Cell survival is normalized with respect to the control submitted to the bleomycin alone. Dead cells are the ones reversibly permeabilized since pulse treatment alone (i.e. without bleomycin) did not induce any cell death (data not shown). For visualization purposes, data points were sometimes slightly laterally shifted.

In order to confirm that the differences observed with one single pulse (Fig. 1) were only due to external conductivity, several other extracellular buffers were tested. The influence of the pH buffering agent for example was tested by preparing three low-conductivity media with sucrose as main component but with three different buffering agents: Tris (STM), Hepes (SHM) and NaHPO4/NaH2PO4 (SNM). Additionally, media using other types of sugars than sucrose were prepared since it is known that sugar type (i.e. sugar size) might influence the outcome of an experiment^23^,^26^. Therefore, adonitol (ATM) and glucose (GTM) were tested. Other high conductivity media were also surveyed: a commercial phosphate buffered saline (PBS) solution as well as two saline solutions prepared in the laboratory that were buffered either with Tris (NTM) or with Hepes (NHM). Other solutions with intermediate conductivity were additionally tested. The detailed compositions of these media are given in the materials and methods section. For all these extracellular media, cells were pulsed in the presence of 30 nM bleomycin, using one single pulse of 100 μs and various electric field magnitudes. All results are summarized on Fig. 3. The graph indicates that reversible permeabilization in high conductivity media (red dashed lines, for σ ≈ 1.5 S/m) required higher field strengths than in intermediate (thick blue dashed lines for σ ≈ 0.6 S/m and thick green lines for σ ≈ 0.375 S/m) and in low conductivity media (thin black lines for σ ≈ 0.1 S/m).

**Figure 4:**
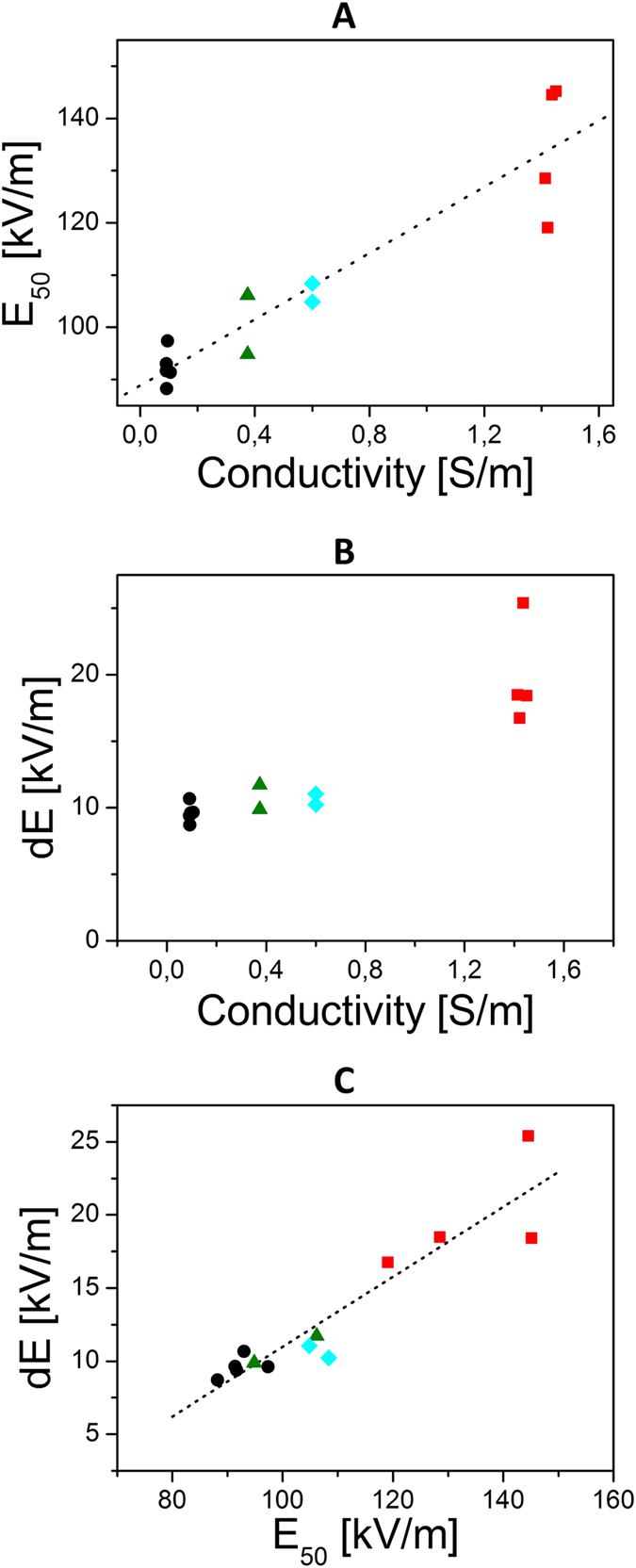
Quantification of the impact of medium's conductivity. (**A**) Electric field at 50% of survival rate as a function of the external medium conductivity. (**B**) Steepness of survival decay as a function of conductivity. (**C**) Steepness of survival decay as a function of the electric field strength at 50% of survival rate. Electric field at 50% of survival rate and steepness of survival decay were extracted from the fit of the data in Fig. 3. Each value of the extracellular medium has been assigned a different symbol: 1.5 S/m (square), 0.6 S/m (diamond), 0.375 S/m (triangles) and 0.1 S/m (circles).

In order to quantify this observation, survival data were fitted by sigmoid functions according to equation (1). The quantity E_50_ corresponds to the electrical field magnitude which is necessary to obtain a survival rate of 50% and dE represents the steepness of the survival decay at E_50_. The quantities E_50_ and dE are plotted as a function of medium’s conductivity on Fig. 4A,B. E_50_ appears to depend linearly on the medium’s conductivity, confirming the fact that reversible permeabilization requires higher field strengths when extracellular conductivity is increased. No impact of the pH buffering agent or sugar type was observed. Additionally, the steepness of survival decay dE (1) was also affected by the extracellular conductivity. For external conductivities ranging between 0.1 S/m and 0.6 S/m, steepness is close to 10 kV/m. Steepness then increases to approximately 20 kV/m for the media with extracellular conductivity of 1.5 S/m. The relation of the steepness dE as a function of E_50_ is displayed on Fig. 4C. It appears that the two quantities are linearly related. Such a dependence indicates that the reversible permeabilisation, that is being quantified, has always the same intrinsic variability as regarded to the field strength.


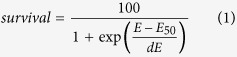


**Figure 5:**
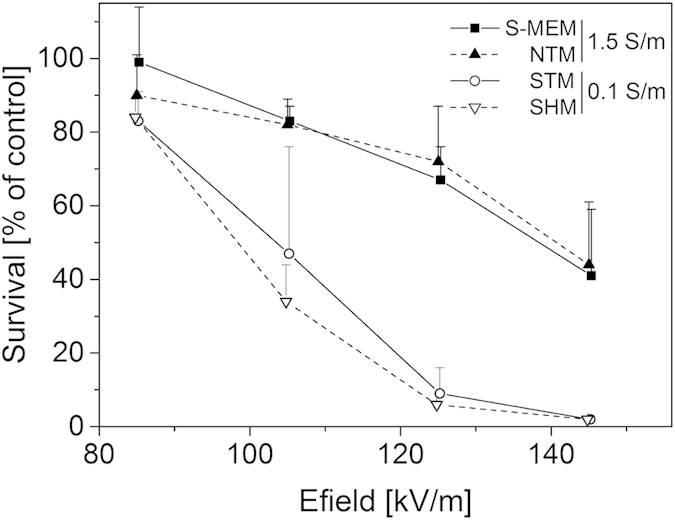
Cell survival as a function of pulse magnitude for different extracellular media. Cloning efficiency tests were performed after exposure to one single micropulse of 100 μs. External medium always contained 30 nM bleomycin. Directly after the delivery of the pulse, cells were diluted in S-MEM medium containing 30 nM bleomycin. Results are represented as mean values + SD of three to six independent experiments. Cell survival is normalized on the control submitted to the bleomycin alone. Dead cells are the ones reversibly permeabilized since pulse treatment alone (i.e. without bleomycin) did not induce any cell death (data not shown).

In all the experiments presented above, cells were kept 10 minutes in their pulsing medium before being handled and diluted for the cloning efficiency assay. In order to check that the ionic strength of the medium did not affect the cells during this 10 minutes period rather than during the pulse delivery, an experiment was performed in which the cells were pulsed in media with different conductivities and immediately diluted in the same highly conductive medium just after the pulses delivery. Only four media were selected for this test: S-MEM (the high-conductivity reference solution; σ = 1.5 S/m), NTM (a high-conductivity saline solution made in the laboratory; σ = 1.5 S/m), STM (the low-conductivity, sucrose based, reference solution; σ = 0.1 S/m) and SHM (a low-conductivity medium similar to STM but buffered with HEPES instead of Tris; σ = 0.1 S/m). Directly after pulse delivery (10 to 20 sec), cells were all diluted in S-MEM solution also containing 30 nM bleomycin so that bleomycin would have time to diffuse in the reversibly permeabilized cells. After a delay of 10 minutes, the standard dilution procedure was performed. Results are displayed on Fig. 5. The experiment was performed with one single pulse of 100 μs. In both high-conductivity media (S-MEM and NTM; both σ = 1.5 S/m) a 50% survival rate is obtained for approximately 145 kV/m while in the two low-conductivity media (STM and SHM; σ = 0.1 S/m) a field value of only 105 kV/m is required i.e. about 30% less. Therefore, the results once more emphasize that a higher field strength was required to obtain reversible permeabilization at high external conductivity.

### Impact of conductivity for pulses in the nanosecond range

In a recent study from our group, performed with the same cell type and the same methodology, the opposite influence of external conductivity was detected when pulses of 12 ns of duration and 3.2 MV/m field strength were used. With those pulse parameters, reversible permeabilization of the cells was obtained only in the high conductivity medium (S-MEM, σ = 1.5 S/m) and not in the low conductivity medium (STM, σ = 0.1 S/m)^22^. In the light of those contradicting observations, a number of supplementary experiments were designed to extend the new data to nanopulses with other pulse parameters. Like in our previous study^22^, the supplementary experiments with nanosecond pulses were restricted to two media, S-MEM and STM.

**Figure 6:**
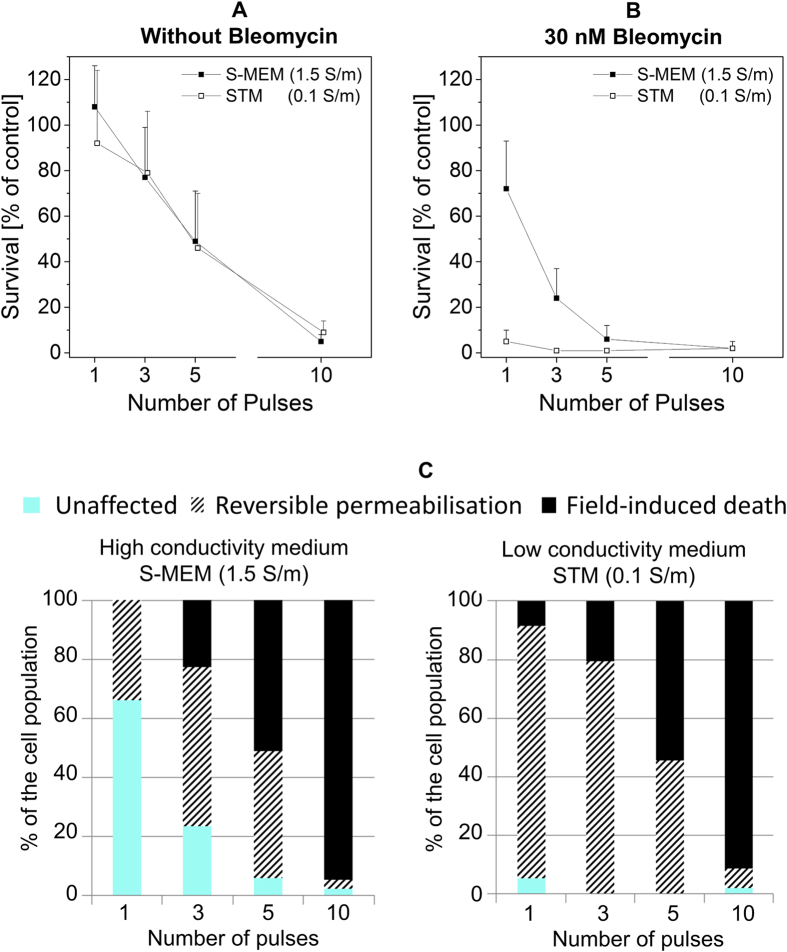
Impact of 12-ns pulses of 14.2 MV/m. Pulses were applied at a repetition rate of 10 Hz. (**A**) Cell viability when cells are exposed in medium alone. (**B**) Cell viability after exposure in medium containing 30 nM of bleomycin. Cell survival is normalized on the control submitted to the bleomycin alone. Results are represented as mean values + SD of three experiments. (**C**) Interpretation of the biological consequences in the two media.

Figure 6 displays the results obtained with pulses of 12 ns of duration and a field magnitude of 14.2 MV/m. At such high magnitude, only a very few number of pulses are required to cause cell death both in the absence and in the presence of bleomycin. When cells were exposed to the pulses in the absence of bleomycin, we observed the same drop of viability in the two media for all the tested conditions (Fig. 6A). In both media, five pulses caused 50% of cell death while 10 pulses provoked the death of more than 90% of the cells. Pulsing the cells in the presence of 30 nM bleomycin resulted however in different viability in the two media (Fig. 6B). In the low conductivity medium, one single pulse resulted already in the death of almost the whole cell population whereas in the high conductivity medium, only 30% of the cells died, and five pulses were required to reach an efficiency similar to that of a single pulse in the low conductivity medium. Figure 6C displays the interpretation of those data in terms of biological consequences. It discriminates the proportion of cells killed just by the exposure to the pulses (referred to as field-induced death) from those reversibly permeabilized i.e. killed only in the presence of bleomycin. This figure clearly illustrates that field-induced death is the same in both the high and the low conductivity media and that, on the contrary, reversible permeabilization is much more efficient in the low conductivity medium (almost up to 100% of the cells upon exposure to a single pulse). These results also demonstrate that field-induced death does not allow to reveal the effects of medium conductivity with these pulse parameters.

**Figure 7:**
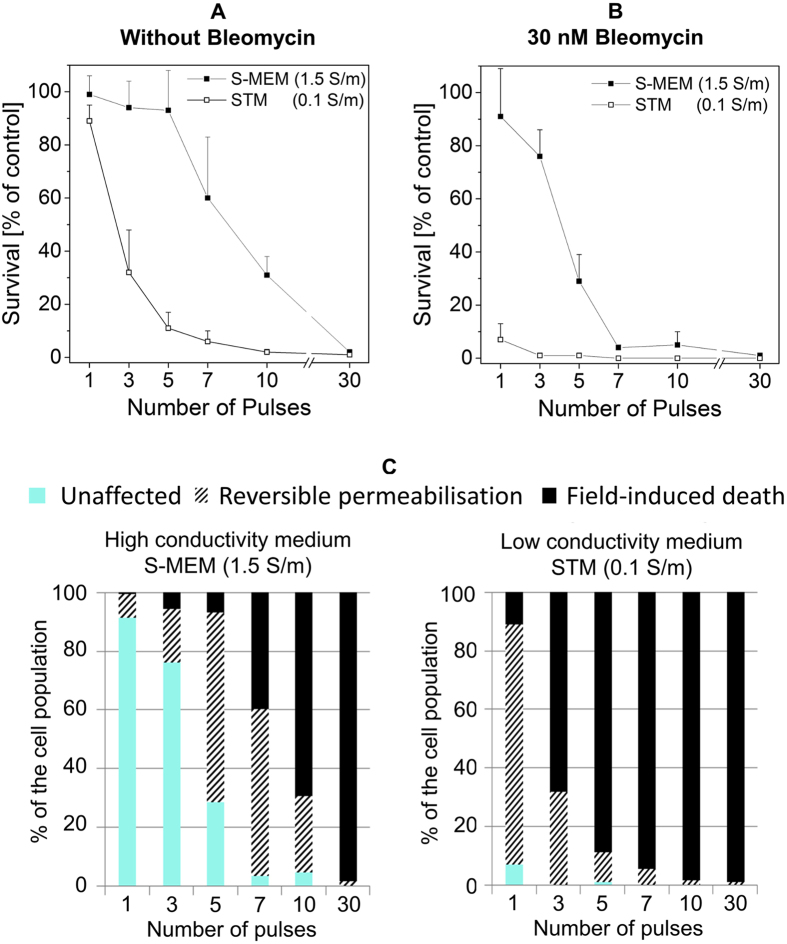
Impact of pulses of 102 ns and 3.2 MV/m. Pulses were applied at a repetition rate of 10 Hz. (**A**) Cell viability when cells are exposed in medium alone. (**B**) Cell viability after exposure in medium containing 30 nM of bleomycin. Cell survival is normalized on the control submitted to the bleomycin alone. Results are represented as mean values + SD of three to six experiments. (**C**) Interpretation of the biological consequences in the two media.

The experiment was repeated again with another set of pulse parameters. The magnitude of the pulses was set to 3.2 MV/m but the duration of the pulses was increased to 102 ns. The repetition rate was kept at 10 Hz. The pulses alone caused different drops in cell survival depending on the media (Fig. 7A). While three pulses provoked no death in the high conductivity medium, they were sufficient to kill approximately 70% of the cells in the low conductivity medium. In order to get the same impact in the high conductivity medium, it was necessary to apply 10 pulses. When 30 nM bleomycin were added to the external medium (Fig. 7B), cell death was already observed at lower number of pulses especially in the low conductivity medium. One single pulse caused the death of almost all the cells exposed in the low conductivity medium. In the high conductivity medium however, viability only started to drop after the application of three pulses, and 10 pulses were necessary to provoke the death of the whole cell population. The viability tests indicate that for those electrical parameters, the low conductivity medium is more efficient to induce both field-induced death and reversible permeabilization (Fig. 7C).

## Discussion

### Experimental evidences of an impact of extracellular conductivity

In the present study, the experiments performed with one single 100 μs-pulse indicate that the efficiency of reversible electropermeabilization significantly increases when the conductivity of the external medium decreases. This result was obtained independently of the individual medium composition. In particular, a possible impact of the pH-buffering agent was excluded. Another critical aspect was that most low-conductivity media usually contain high concentration of sugars (like sucrose) which can prevent the typical cell swelling which is observed when pulses are applied in the standard saline solution^26^. The possible impact of the pulse-induced swelling was tested by replacing sucrose with adonitol, a polyol that does not prevent swelling^26^: results suggest that cell swelling, a post-pulse event, does not play a role. The radius of the cells was also controlled in the majority of the buffer used and while overall, some small changes of size could be observed (see material and method section), no clear correlation between size and cell survival could be extracted.

The impact of extracellular conductivity at the time of the pulse delivery was preserved even when cells were resuspended in their culture medium a few seconds after the delivery of the electric pulses suggesting that the differences observed were not due to the post-pulse incubation conditions. A higher efficiency of low conductivity media was also observed when using a few 12-ns or 102-ns pulses (14.2 and 3.2 MV/m, respectively), or just a single pulse of these lengths. The results presented in this study are thus overall consistent since the same tendency was obtained for many different pulse parameters.

These results are in agreement with the studies from the group of Zimmermann who performed experiments with ‘exponential pulses’ in the microsecond range^17^,^18^,^19^ and also with square pulses ranging from 10 to 100 ns^8^. Our results however contradict some of the previously published data indicating that the permeabilisation efficiency positively correlated with external conductivity or that there was no influence of the external medium conductivity^13^,^14^,^15^,^16^ including one of our recent studies^22^.

Some of the apparent contradictions between all the different studies can be accounted for by at least two facts. First of all, the various studies suggest that the influence of the conductivity can be different depending on whether reversible permeabilization or pulses-induced cell-death is considered. This has been reported in previous publications^14^,^17^ and can also been seen in this study. Indeed, the results obtained with pulses of 12 ns and 14.2 MV/m indicate that extracellular conductivity does not influence field-induced cell-death (Fig. 6A) while it has a marked influence on reversible permeabilisation efficiency (Fig. 6B). This observation can be explained by the fact that the cell-death induced by electric pulses can occur through various mechanisms^23^ and is strongly influenced by the composition of the medium^17^,^27^. Detection methods which are unspecific and/or which cannot discriminate between reversible permeabilisation and pulse-induced cell death should therefore be avoided since effects of medium composition may dominate the effect of conductivity and thus complicate the results interpretation. Additionally, it appears that the influence of extracellular conductivity might not be visible for some specific pulse parameters. In the study of Pucihar and colleagues, no influence of conductivity on reversible permeabilisation could be detected on DC3-F cells when 8 pulses of 100 μs length were applied^14^. We have reproduced this experiment (Fig. 2) and fully agree with the conclusions of our colleagues. However, we have shown an important effect of conductivity when only one pulse of 100 μs was applied. Most likely, the absence of impact of conductivity that is observed with 8 pulses is the consequence of a saturation phenomenon. Indeed, it is known that the accumulation of pulses increases the permeabilization efficiency until a saturation is reached. This has been demonstrated on CHO cells in suspension which are similar in size^28^ to the DC3-F cells. Saturation of permeabilisation-induced uptake of Direct-blue^28^ was detected after accumulation of 3 pulses of 100 μs (100 kV/m) and saturation of the release of ATP^29^ after 10 pulses of 100 μs (160 kV/m). Therefore, assumption of a saturation of bleomycin uptake after 8 pulses of 100 μs and 80 kV/m appears reasonable. The detailed mechanisms of saturation are beyond the scope of this paper. However a simple explanation is that pulse magnitude defines the area of the cell which is permeabilized while pulse accumulation solely increases the intensity of permeabilisation of that area (e.g. by increasing the number of pores like recently suggested for 60-ns pulses^30^) until a saturation of the damages on that area is reached (e.g. a maximum pore density that cannot be overcome).

The fact that a saturation effect is able to mask the influence of the external medium conductivity was also revealed by the influence of the bleomycin concentration on the results of our experiments. Indeed, in Fig. 1, the impact of extracellular conductivity was more visible in the presence of 5 nM bleomycin than in the presence of 30 nM bleomycin. All these data highlight the difficulty to analyze cell membrane permeabilization by means of one of its consequences, i.e. the transport of molecules across the permeabilized membranes. The issue of “saturating conditions” in the domain of the cell membrane permeabilization was already well described in the literature, concerning not only the number of pulses but also the duration of the pulses^29^,^31^. Moreover, the pulse parameters that induce saturation depend on the detection method employed, for example on the type of dye used to detect transport across the membrane^31^ or on the concentration of the dye that is used, like in our case for the bleomycin concentration. Such difficulties might explain why sometimes no influence of the extracellular conductivity has been detected.

The numerous factors that might influence a permeabilisation experiment also imply that the results which were obtained in this study with bleomycin might not hold when other markers of permeabilisation are used. Preliminary experiments carried in our lab indicate that our conclusions on the impact of extracellular conductivity on reversible permeabilisation hold when YO-PRO is used and at least one of the previous published study which focused specifically on reversible permeabilisation also indicate similar conclusions with PI used as a marker^17^. In the specific case of PI, it has been suggested that extracellular conductivity might have an influence not only of permeabilisation but also on the electrophoretic transport of propidium since it can be enhanced at low conductivity^32^. In the case of bleomycin, no influence of the conductivity on the transport is expected since transport occurs almost exclusively by diffusion and is not transported by electrophoresis^33^. However, a thorough study using different markers would re-inforce the present data and enable to draw larger conclusion regarding the impact of extracellular conductivity on permeabilisation.

Among the different papers specifically focusing on the effect of extracellular conductivity on reversible permeabilisation, the only one contradicting the conclusion of the present study, to the best of our knowledge, is therefore the one from our group^22^, recently published, in which we indeed reported the opposite tendency. With the same experimental approach, on the same cell-type, we have shown that pulses of 12 ns and 3.2 MV/m could induce reversible permeabilization when the external conductivity was 1.5 S/m (S-MEM medium) while no effect was detected when the external conductivity was 0.1 S/m (STM medium). Therefore, even when only reversible permeabilisation is considered and even in the absence of any saturation phenomenon, influence of conductivity appears to depend on pulse parameters.

**Figure 8:**
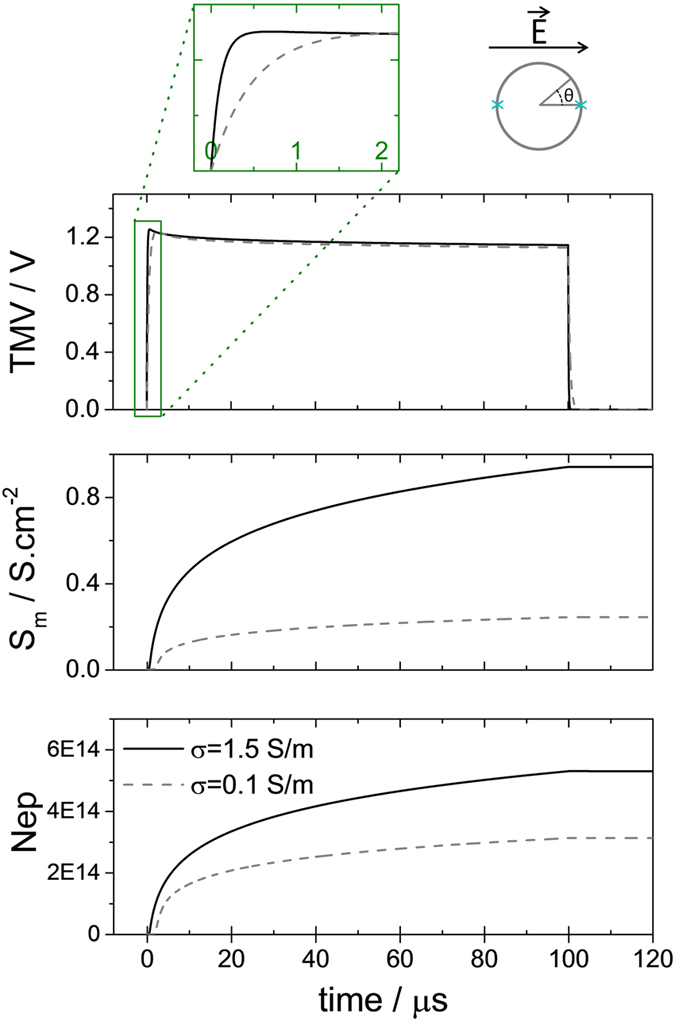
Simulation of the electroporation process for a spherical cell exposed to a 100 μs pulse of 105 kV/m and for two values of external conductivities. Simulation was performed using the model of Krassowska and colleagues. Resting transmembrane voltage has been neglected. (Top) Time course of the transmembrane voltage V at the pole of a spherical cell. The pole correspond to θ = 0 [π] as depicted on the schematic. The additional insert displays a zoom of the first microseconds (Center) Time course of membrane conductance increase at the pole. (Bottom) Time course of the pore density at the pole.

In the specific contradicting case of the 12 ns and 3.2 MV/m pulses, the efficiency of the pulses was however limited since with an external bleomycin concentration of 30 nM, 100 pulses were necessary to detect a bleomycin-induced cell death in 60% of the cell population in a high-conductivity medium i.e. S-MEM (Fig. 4 in reference^12^). The extremely low permeabilising effect of only one of those pulses in S-MEM could be detected but it required to increase the external concentration of bleomycin to 3 μM^12^. In low-conductive medium like STM, 1000 pulses did not enable bleomycin uptake or at least not enough to induce cell-death. It is therefore reasonable to assume that those pulses, induce no membrane conductivity increase in low-conductive medium like STM and a negligible membrane conductivity increase in high-conductivity medium like S-MEM. The linear dielectric model describing membrane charging in the absence of changes of membrane conductivity and which is based only on electromagnetic equations, namely the Kirchhoff law, can therefore be used to compute the transmembrane voltage (TMV) induced by external field. This models predicts that the TMV induced by a pulse of 12 ns and 3.2 MV/m on a DC3-F cells increases by approximately a factor four (from 1 V to 4 V) when conductivity increases from 0.1 to 1.5 S/m (as depicted in Fig. 8 in reference^22^). This difference arises from the fact that membrane charging-time *τ* strongly depends on extracellular conductivity (equation 2) and typically increases by a factor 4 when the extracellular conductivity decreases from 1.5 S/m to 0.1 S/m. Therefore, if one assumes that the value of the TMV is the main trigger of electropermeabilisation, the linear dielectric model predict that the 12 ns pulses of 3.2 MV/m will be more efficient in the high-conductivity medium even if, as discussed, the efficiency is very limited.


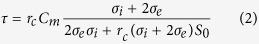


*r*_*c*_ *cell radius; C*_*m*_ *membrane surface capacitance (F/m*^*2*^*); σ*_*i*_ *and σ*_*e*_ *intracellular and extracellular conductivities (S/m); S*_*0*_ *membrane surface conductance (S/m*^*2*^).

For all the parameters tested in the present study, pulses were efficient at inducing reversible permeabilisation in the sense that uptake of bleomycin (with an external concentration of 30 nM) could be detected after either a single pulse or a very low number of pulses. In that case, the linear model of the TMV cannot be used anymore since membrane conductivity increase must be accounted for. Figure 8 presents simulation of the impact of a pulse of 100 μs of 105 kV/m on a DC3-F cell for two values of extracellular conductivity using the most popular model i.e the one developed by Krassowska and colleagues^34^. The TMV, the membrane conductance and the pore density at the pole of the cell (θ = 0, as depicted in the inset) are represented. The parameters of the model are given in Table 1 of reference^34^, except for cell radius which equals 7.5 μm for DC3-F cells, intracellular conductivity which is 1 S/m and extracellular conductivity which is either 0.1 S/m or 1.5 S/m as in our experiments.

As depicted on Fig. 8 (top) the model predicts that the final value of the TMV during a 100 μs-long pulse is almost independent of the extracellular medium conductivity and it in fact depends only on the pulse amplitude as for the linear model. An impact of the external conductivity of the TMV can only be observed during the first microseconds i.e. during the charging period (Fig. 8 top, zoom). After approximately 2 μs the TMV is stabilized until the end of the pulse at the same value for both extracellular conductivities. Additionally, the membrane conductance (Fig. 8 middle), and the pore density (Fig. 8 bottom) are higher in the high conducting medium, in contradiction with our experimental observations. Note that similar numerical results are obtained with the model of Leguèbe Poignard *et al.*^35^,^36^. The main reason for which the different existing models do not corroborate our experimental data lies in the fact that the medium conductivity influences pore creation only indirectly through the Kirchhoff law while pore creation rate, or more generally speaking membrane conductance increase, depends only on the instantaneous value of the TMV. Therefore according to the models, during the first microsecond of the pulse, more pores are created in the higher conductive medium, since the TMV is higher. After 1 μs the TMV reaches the same steady state, which does not depend of the medium conductivity and therefore the same number of pores are created at any time after 1us in both media. This eventually leads to a higher membrane conductance in the high conductive medium at the end of the pulse, which is in contradiction with the data. Similar results can be obtained with shorter pulses of 10 ns or 100 ns for which the different models systematically predict a higher efficiency of permeabilisation for higher values of extracellular conductivities.

### Hypothesis regarding the role of extracellular conductivity

At the current state of our work, we cannot provide a unique explanation for the impact of the extracellular conductivity. Most probably, extracellular conductivity influences electropermeabilisation in more than one way. As it was detailed in our previous paper^22^ and recalled above, membrane charging probably plays a role and can alone explain the specific cases in which membrane conductance is weakly increased. In all other cases, we can only speculate on different possible explanations/hypothesis to describe the role of conductivity:

1. Our first hypothesis is a purely electrical explanation. As described above, the current models of electroporation cannot account for the impact of conductivity. Their main drawback is that they consider the membrane as a continuum and cannot account for the local behavior on a nanoscopic scale. If a high TMV is assumed to create pores of a few nanometers, conductance of ion through such pores will locally^37^ decrease the TMV and in turn lead to pore shrinking or pore closure. By preventing pore expansion or pore stabilization, the local drop of TMV at the level of the pore might act therefore as a protection for the membrane. The partial closure of the pores when the TMV decreases has been modeled in Molecular Dynamics simulations and supports this hypothesis^38^. Intuitively, local TMV drop across a pore due to ion conduction should be faster in a medium of high conductivity since it contains more charge carriers. This is equivalent in saying that the conductivity of a pore will depend on the conductivities of intracellular and extracellular media as it was already suggested^39^. This however remains to be tested, the most plausible approach being molecular dynamics. It also remains to be figured out how a very local effect at the level of pore can be incorporated in models which describe the membrane as a continuum.
2. Another hypothesis is based on electromechanical coupling. In the presence of an electric field, Maxwell stress induces a stretching force on the membrane^40^,^41^,^42^. This force is higher at lower extracellular conductivity as predicted by equations and demonstrated experimentally^18^. This was proposed by Zimmermann and colleagues who observed a higher efficiency of electropermeabilisation in low conductivity medium with exponential microsecond-pulses and square nanosecond-pulses^8^,^17^,^18^. One should note that even if a mechanical force is applied, the cell will not necessarily undergo electric-field-induced deformation like it has been reported for GUVs^43^. Indeed, the reported kinetics of those deformations are rather slow, needing times longer than the micropulses duration and in fact, to the best of our knowledge, electro-deformation of cells under pulsed electric field has also not been reported so far. However, the stretching forces along the field lines are established in a couple of nanoseconds^18^ and generate a tension on the membrane even if global deformation does not occur. Moreover it is not excluded that Maxwell stress can induce significant deformation on the nanometer scale within pulse duration^44^. Since membrane tension is known to influence permeabilisation thresholds^45^, it would translate in our experiment into an influence of the conductivity.
3. Finally, the impact of the conductivity might not be truly during the pulse but external medium conductivity might affect the initial state of the membrane before the pulse. It is known for example that ionic strength and sugar content have an impact on membrane properties such as bending rigidity and stretching coefficient^46^. Those properties of the membrane are known to directly affect permeabilization thresholds^45^ and the fact that stiffer membranes are more resistant to pulsed electric fields was even demonstrated on red blood cells^19^. We have been able to exclude an influence of the type of sugar and of the type of buffer, but the characterization of the biophysical parameters of the cell membrane in the various media used in our study requires further investigations.

## Conclusion

It is currently not possible to explain precisely the influence of conductivity on electropermeabilisation. In any case the results demonstrate that electropermeabilisation efficiency cannot be predicted by the current electroporation models and also does not simply scales on macroscopic electrical parameters like magnitude of the electric field, current density or global energy delivered to the sample. Only microscopic parameters, electrical and mechanical, at the level of the membrane can fully describe the phenomenon. This is important for our understanding of cell membrane electroporation but has also some direct practical consequences for electroporation-based applications. Indeed, energy expenditure generated by electroporation treatment can sometimes be a hindrance to the development of certain applications. This is especially true for applications in the field of energy such as microalgae based biorefinary^47^,^48^. Reducing the conductivity of the extracellular medium before treatment by electric pulses is an approach that would greatly reduce energy costs while maintaining or even improving the efficiency of the treatment.

In conclusion, we would like to emphasize that the effect of medium conductivity has been detected because in most of our experiments only one single electric pulse was delivered to the cells. The experiments performed with 100 μs pulses were initially performed using one single pulse in order to avoid the complex analysis of the influence of the number of the pulses as well as of their repetition rate^49^,^50^. In the course of our experiments, it has been striking to note that the influence of the medium conductivity, that is detected when one single pulse of 100 μs is applied, becomes undetectable when eight pulses are delivered. This result highlights the importance of the delivery of one single pulse in the electroporation analytical studies.

## Material and Methods

### Cell culture

**Figure 9:**
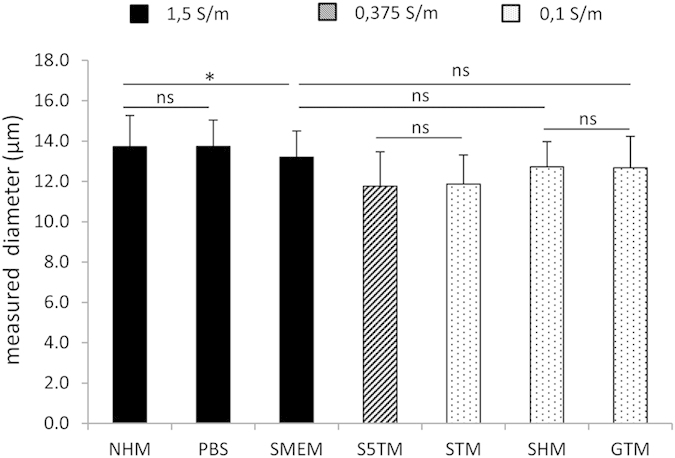
Average diameter of the DC3-F cells in suspension in different media. Data are presented as mean + SD of at least 100 individual cells. Statistical differences were analyzed using the non-parametric Kruskall-Wallis test with Dunn’s Multiple Comparison test. For clarity, only low significant differences (*0.01 < p < 0.05) and non-significant differences (ns, p > 0.05) have been indicated.

Chinese hamster lung cell line DC-3F [24] was grown in complete medium which consists in MEM-Minimum Essential Medium (31095-052, Life Technologies, Saint Aubin, France) with addition of 10% fetal bovine serum (10270-106, Life Technologies) and supplemented by antibiotics (500 U/ml penicillin, and 500 μg/ml streptomycin). The cells were maintained in a humidified atmosphere at 37˚C and 5% CO2 and routinely sub-cultured every two days. When they are placed in suspension, those cells have a spherical shape. The average diameter (mean ± standard deviation) was evaluated from bright light microscopy images in different extracellular medium (Fig. 9). While the size of the cells could vary slightly depending on the medium, it showed no correlation with the conductivity of the medium.

### Pulsing media

The composition, the osmolarity and the conductivity of all the media used are described in Table 1. Two media are commercially available from Life Technologies: S-MEM (11380-037) and PBS (14200–083). All other media were prepared in the laboratory and only contained one or two compounds (sugars, salts) to control the osmolarity, a buffer to control the pH, and 1 mM MgCl_2_ (Table 1). Three different types of sugar were used to prepare the low conductivity media: sucrose (S), adonitol (A) and glucose (G). To prepare the high conductivity media, NaCl replaced the sugar and the intermediate conductive media contained a mix of sugar and NaCl. Three buffering agents were tested: Tris (T), Hepes (H) and NaHPO4/NaH2PO4 (N). The pH was adjusted to 7 by addition of HCl and conductivity was adjusted by adding NaCl (concentrations are given in Table 1). All products were purchased from Sigma Aldrich or from Life Technologies (Hepes and PBS).

Conductivities of the solutions were measured with a Conductivity Meter CLM 381 (Endress and Hauser, Weil am Rhein, Germany) and osmolarities of the solutions were measured with a vapor pressure osmometer Vapro 5600 (ELITechGroup Wescor, South Logan, Utah, USA).

The media containing bleomycin were prepared using 300 μM bleomycin stock solutions obtained by diluting lyophilized bleomycin in S-MEM or STM. Stock solutions were stored at −20 °C. Aliquots were taken just before the experiments and dissolved in the appropriate medium to obtain media with 30 nM bleomycin final concentration. The stock solution in S-MEM was used for all the media with a 1.5 S/m conductivity and the stock solution in STM for all the other media.

### Assessment of cell viability

After trypsinization of exponentially growing cells and inactivation of trypsin (25300-054, Life Technologies) by the serum factors of the complete medium, cells were centrifuged for 10 min at 150 g and resuspended at a density of 4.2 × 10^6^ cells/ml in the appropriate medium with or without bleomycin (final bleomycin concentration was either 5 nM or 30 nM). Cells were immediately deposited between the two electrodes and exposed to the electric pulses. Unless otherwise specified, cells were kept for 10 min at room temperature after the delivery of the electric pulses. They were then diluted in complete medium. After dilution, cells were seeded in triplicate in complete culture medium (250 cells per cell culture dish, 35 mm in diameter) to measure their viability through a quantitative cloning efficacy test. After 5 days of culture at 37 °C in a humidified, 5%CO2 incubator, colonies were fixed and stained (with a solution of 3.7 % formaldehyde containing crystal violet) and the number of clones N for each condition was counted. Viability was then normalized to the number of clones in the control N_control_ and reported as a percentage of survival: N/N_control_.10^2^. In experiments without bleomycin, the control condition refers to cells submitted to no treatment. In experiments with bleomycin, the control condition refers to cells, in contact with the bleomycin, that did not receive pulses. Viability of unpulsed controls was typically between 90% and 95 % after exposure to 30 nM bleomycin only.

### Exposure of cells to pulsed electric field

Cells were exposed in suspension in conventional electroporation cuvettes from Cell project (Harrietsham, United Kingdom). Depending on the experiment, 1 mm or 4 mm cuvettes were used. Precise measurements of the distance between the electrodes indicated that the distance between the electrodes was d_1mm_ = 1.22 ± 0.02 mm for the 1 mm cuvettes and d_4mm_ = 4.19 ± 0.02 mm for the 4 mm cuvettes. Those measured distances were the ones considered when the inter-electrode distance was needed to evaluate the field inside the cuvette. During experiments with 100 μs-pulses, the 1 mm cuvettes were used and filled with 60 μL of cell suspension.

During the experiments designed to test the influence of conductivity on the efficiency of nanosecond pulses, special care was taken to impose the exact same electric field in both the high and low conductivity medium. This can be difficult due to reflection phenomena that may occur^51^. In order to overcome this problem, the two cuvettes containing the two different media were exposed in parallel (this was already detailed in^22^). The volumes of cell suspension inside the cuvette were chosen in order to limit the reflection of the pulse at the level of the sample through the matching of the load impedance to the circuit and generator impedance. In order to avoid electrical breakdown, the remaining space between the electrodes was filled with paraffin oil (Sigma-Aldrich, Saint-Louis, MO, US). For the two types of nanosecond pulses that were considered, the cuvette type and the volumes of medium per cuvette were chosen according to Table 2.

### Pulses generator and measurement

Pulses of 100 μs duration (micropulses) were delivered with a repetition rate of 1Hz by an electroporation power supply (CliniporatorTM, IGEA, Carpi, Italy) able to apply high-voltage square-wave pulses. Voltage waveforms were controlled and recorded with an oscilloscope (LeCroy WaveMaster 808Zi) and a voltage probe (LeCroy PP007-WR).

**Figure 10:**
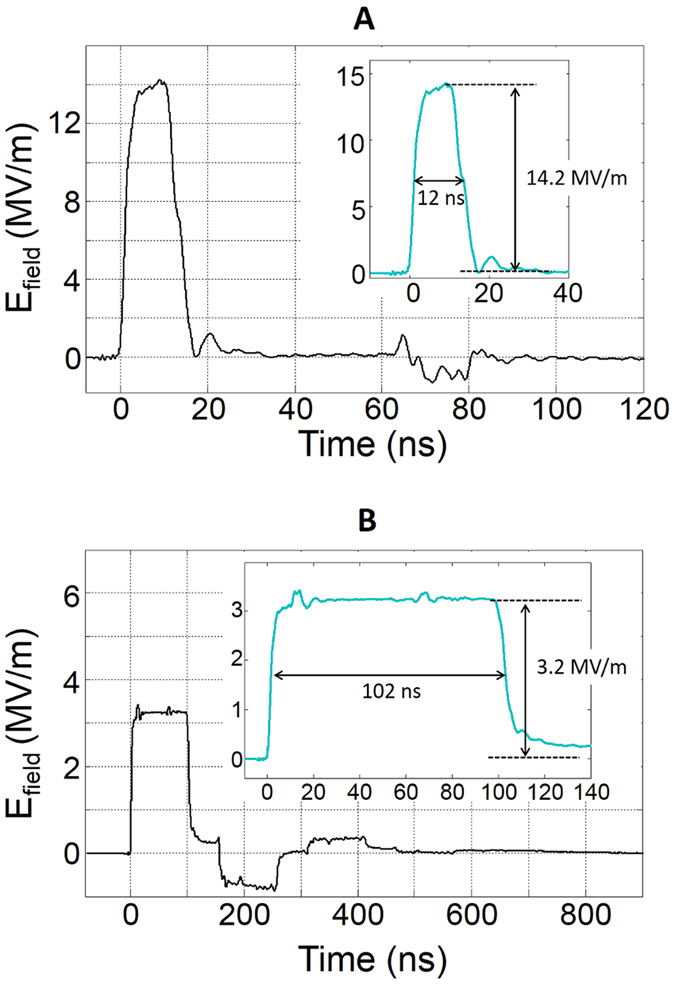
The nanosecond pulses applied to the samples. (**A**) Pulse of 14.2 MV/m and 12 ns (**B**) Pulse of 3.2 MV/m and 102 ns.

Pulses of 12 ns and 102 ns were delivered by a pulse-forming line generator, based on spark gap technology and designed by Europulse (Cressensac, France). Different interchanging cables were used to obtain the different pulse durations. Voltage measurements were performed using the D-dot sensor which has been described in detail in references^22^ and^52^. The real pulses applied on the sample are displayed on Fig. 10. As can be seen, the 12-ns pulse is almost free of post-pulse rebound. Conversely, the 102-ns pulse displays a rebound just after the main pulse. An absence of rebound would be preferable but the appropriate matching conditions could not be obtained. We stress however that samples with different conductivities were exposed simultaneously and were therefore submitted to exactly the same pulse with the same rebound.

## Additional Information

**How to cite this article**: Silve, A. *et al.* Impact of external medium conductivity on cell membrane electropermeabilization by microsecond and nanosecond electric pulses. *Sci. Rep.* **6**, 19957; doi: 10.1038/srep19957 (2016).

## Figures and Tables

**Table 1:** Composition and properties of the different media.

Buffer	Sugar	MgCl_2_	NaCl	PH Buffer	σ [S/m]	osm [mOsm]
STM	Sucrose/50	1	–	Tris/10	0.096	287
ATM	Adonitol/250	1	–	Tris/10	0.093	280
GTM	Glucose/250	1	–	Tris/10	0.106	274
SNM	Sucrose/250	1	5	Na_2_HPO_4_/5 - NaH_2_PO_4_/5	0.092	274
SHM	Sucrose/250	1	6	Hepes/10	0.092	284
S5HM	Sucrose/210	1	30	Hepes/50	0.375	351
S5TM	Sucrose/210	1	–	Tris/50	0.375	318
NSHM	Sucrose/110	1	74	Hepes/10	0.600	275
NSTM	Sucrose/110	1	70	Tris/10	0.600	283
NHM	–	1	140	Hepes/10	1.450	263
NTM	–	1	140	Tris/10	1.421	283
**PBS**	*cf commercial technical ressource*				1.436	295
**SMEM**	*cf commercial technical ressource*				1.413	288

Concentrations are given in mM. pH was adjusted to 7 by addition of HCl.

**Table 2:** Experimental conditions for the delivery of nanosecond pulses at identical field amplitudes. For each condition, two cuvettes were exposed in parallel.

Pulse type	Cuvette type	Volumes per cuvette
12 ns 14.2 MV/m	1 mm	25 μL of cell suspension 120 μL of paraffin oil
102 n 3.2 MV/m	4 mm	400 μL of cell suspension 600 μL of paraffin oil

In one cuvette cells were in the low conductivity medium and in the second one in the high conductivity medium.

## References

[b1] Mir L. M., Banoun H. & Paoletti C. Introduction of definite amounts of nonpermeant molecules into living cells after electropermeabilization: Direct access to the cytosol. Exp. Cell Res. 175, 15–25 (1988). 3345798 10.1016/0014-4827(88)90251-0

[b2] Neumann E. Membrane electroporation and direct gene transfer. Bioelectrochem. Bioenerg. 28, 247–267 (1992).

[b3] Teissie J., Golzio M. & Rols M. P. Mechanisms of cell membrane electropermeabilization: a minireview of our present (lack of ?) knowledge. Biochim. Biophys. Acta 1724, 270–280 (2005). 15951114 10.1016/j.bbagen.2005.05.006

[b4] Mir L. M. Therapeutic perspectives of *in vivo* cell electropermeabilization. Bioelectrochemistry Amst. Neth. 53, 1–10 (2001). 10.1016/s0302-4598(00)00112-4 11206915

[b5] Silve A. & Mir L. M. in Clinical Aspects of Electroporation (eds. Kee S. T., Gehl J. & Lee E. W.) 69–82 (Springer: New York, , 2011). at < http://www.springerlink.com.gate1.inist.fr/content/r86178281342200g/>.

[b6] Mir L. M. Nucleic acids electrotransfer-based gene therapy (electrogenetherapy): past, current, and future. Mol. Biotechnol. 43, 167–176 (2009). 19562526 10.1007/s12033-009-9192-6

[b7] Sack M. *et al.* Research on Industrial-Scale Electroporation Devices Fostering the Extraction of Substances from Biological Tissue. Food Eng. Rev. 2, 147–156 (2010).

[b8] Müller K. J., Sukhorukov V. L. & Zimmermann U. Reversible electropermeabilization of mammalian cells by high-intensity, ultra-short pulses of submicrosecond duration. J. Membr. Biol. 184, 161–170 (2001). 11719852 10.1007/s00232-001-0084-3

[b9] Pakhomov A. G. *et al.* Lipid nanopores can form a stable, ion channel-like conduction pathway in cell membrane. Biochem. Biophys. Res. Commun. 385, 181–186 (2009). 19450553 10.1016/j.bbrc.2009.05.035 PMC2739132

[b10] Vernier P. T., Sun Y. & Gundersen M. A. Nanoelectropulse-driven membrane perturbation and small molecule permeabilization. BMC Cell Biol. 7, 37 (2006). 17052354 10.1186/1471-2121-7-37 PMC1624827

[b11] Vernier P. T., Sun Y., Chen M.-T., Gundersen M. A. & Craviso G. L. Nanosecond electric pulse-induced calcium entry into chromaffin cells. Bioelectrochemistry Amst. Neth. 73, 1–4 (2008). 10.1016/j.bioelechem.2008.02.003 18407807

[b12] Silve A., Leray I. & Mir L. M. Demonstration of cell membrane permeabilization to medium-sized molecules caused by a single 10ns electric pulse. Bioelectrochemistry Amst. Neth. (2011), doi: 10.1016/j.bioelechem.2011.10.002. 22074790

[b13] Neumann E. Membrane electroporation and direct gene transfer. Bioelectrochem. Bioenerg. 28, 247–267 (1992).

[b14] Pucihar G., Kotnik T., Kanduser M. & Miklavcic D. The influence of medium conductivity on electropermeabilization and survival of cells *in vitro*. Bioelectrochemistry Amst. Neth. 54, 107–115 (2001). 10.1016/s1567-5394(01)00117-7 11694390

[b15] Ferreira E. *et al.* Optimization of a gene electrotransfer method for mesenchymal stem cell transfection. Gene Ther. 15, 537–544 (2008). 18256695 10.1038/gt.2008.9

[b16] Antov Y., Barbul A., Mantsur H. & Korenstein R. Electroendocytosis: Exposure of Cells to Pulsed Low Electric Fields Enhances Adsorption and Uptake of Macromolecules. Biophys. J. 88, 2206–2223 (2005). 15556977 10.1529/biophysj.104.051268 PMC1305271

[b17] Djuzenova C. S. *et al.* Effect of medium conductivity and composition on the uptake of propidium iodide into electropermeabilized myeloma cells. Biochim. Biophys. Acta 1284, 143–152 (1996). 8914578 10.1016/s0005-2736(96)00119-8

[b18] Sukhorukov V. L., Mussauer H. & Zimmermann U. The effect of electrical deformation forces on the electropermeabilization of erythrocyte membranes in low- and high-conductivity media. J. Membr. Biol. 163, 235–245 (1998). 9625780 10.1007/s002329900387

[b19] Mussauer H., Sukhorukov V. L., Haase A. & Zimmermann U. Resistivity of Red Blood Cells Against High-Intensity, Short-Duration Electric Field Pulses Induced by Chelating Agents. J. Membr. Biol. 170, 121–133 (1999). 10430656 10.1007/s002329900542

[b20] Ivorra A., Villemejane J. & Mir L. M. Electrical modeling of the influence of medium conductivity on electroporation. Phys. Chem. Chem. Phys. 12, 10055 (2010). 20585676 10.1039/c004419a

[b21] Schoenbach K., Joshi R., Beebe S. & Baum C. A scaling law for membrane permeabilization with nanopulses. IEEE Trans. Dielectr. Electr. Insul. 16, 1224–1235 (2009).

[b22] Silve A., Leray I., Leguèbe M., Poignard C. & Mir L. M. Cell membranes permeabilization by 12-ns electric pulses: Not a purely dielectric, but a charge-dependent phenomenon. Bioelectrochemistry, doi: 10.1016/j.bioelechem.2015.06.002. 26138342

[b23] Pakhomova O. N., Gregory B. W., Semenov I. & Pakhomov A. G. Two modes of cell death caused by exposure to nanosecond pulsed electric field. PloS One 8, e70278 (2013). 23894630 10.1371/journal.pone.0070278 PMC3720895

[b24] Morotomi-Yano K., Akiyama H. & Yano K. Different involvement of extracellular calcium in two modes of cell death induced by nanosecond pulsed electric fields. Arch. Biochem. Biophys. 555-556, 47–54 (2014). 24893145 10.1016/j.abb.2014.05.020

[b25] Poddevin B., Orlowski S., Belehradek J. Jr & Mir L. M. Very high cytotoxicity of bleomycin introduced into the cytosol of cells in culture. Biochem. Pharmacol. 42 Suppl, S67–75 (1991). 1722669 10.1016/0006-2952(91)90394-k

[b26] Nesin O. M., Pakhomova O. N., Xiao S. & Pakhomov A. G. Manipulation of cell volume and membrane pore comparison following single cell permeabilization with 60- and 600-ns electric pulses. Biochim. Biophys. Acta 1808, 792–801 (2011). 21182825 10.1016/j.bbamem.2010.12.012 PMC3039094

[b27] Baldwin W. H., Gregory B. W., Osgood C. J., Schoenbach K. H. & Kolb J. F. Membrane Permeability and Cell Survival After Nanosecond Pulsed-Electric-Field Exposure #x02014;Significance of Exposure-Media Composition. IEEE Trans. Plasma Sci. 38, 2948–2953 (2010).

[b28] Gabriel B. & Teissié J. Control by electrical parameters of short- and long-term cell death resulting from electropermeabilization of Chinese hamster ovary cells. Biochim. Biophys. Acta BBA - Mol. Cell Res. 1266, 171–178 (1995). 10.1016/0167-4889(95)00021-j 7742383

[b29] Rols M. P. & Teissié J. Electropermeabilization of mammalian cells. Quantitative analysis of the phenomenon. Biophys. J. 58, 1089–1098 (1990). 2291935 10.1016/S0006-3495(90)82451-6 PMC1281055

[b30] Pakhomov A. G. *et al.* Multiple nanosecond electric pulses increase the number but not the size of long-lived nanopores in the cell membrane. Biochim. Biophys. Acta BBA - Biomembr. 1848, 958–966 (2015). 10.1016/j.bbamem.2014.12.026 PMC4331219 25585279

[b31] Rols M.-P. & Teissié J. Electropermeabilization of Mammalian Cells to Macromolecules: Control by Pulse Duration. Biophys. J. 75, 1415–1423 (1998). 9726943 10.1016/S0006-3495(98)74060-3 PMC1299816

[b32] Li J. & Lin H. Numerical simulation of molecular uptake via electroporation. Bioelectrochemistry 82, 10–21 (2011). 21621484 10.1016/j.bioelechem.2011.04.006

[b33] Sungailaitė S., Ruzgys P., Šatkauskienė I., Čepurnienė K. & Šatkauskas S. The dependence of efficiency of transmembrane molecular transfer using electroporation on medium viscosity. J. Gene Med. 17, 80–86 (2015). 25761762 10.1002/jgm.2825

[b34] DeBruin K. A. & Krassowska W. Modeling electroporation in a single cell. I. Effects Of field strength and rest potential. Biophys. J. 77, 1213–1224 (1999). 10465736 10.1016/S0006-3495(99)76973-0 PMC1300413

[b35] Kavian O., Leguèbe M., Poignard C. & Weynans L. ‘Classical’ electropermeabilization modeling at the cell scale. J. Math. Biol. 68, 235–265 (2014). 23239007 10.1007/s00285-012-0629-3

[b36] Leguèbe M., Silve A., Mir L. M. & Poignard C. Conducting and permeable states of cell membrane submitted to high voltage pulses: mathematical and numerical studies validated by the experiments. J. Theor. Biol. 360, 83–94 (2014). 25010659 10.1016/j.jtbi.2014.06.027

[b37] Winterhalter M. & Helfrich W. Effect of voltage on pores in membranes. Phys. Rev. A 36, 5874–5876 (1987). 10.1103/physreva.36.5874 9898883

[b38] Fernández M. L., Risk M., Reigada R. & Vernier P. T. Size-controlled nanopores in lipid membranes with stabilizing electric fields. Biochem. Biophys. Res. Commun. 423, 325–330 (2012). 22659739 10.1016/j.bbrc.2012.05.122

[b39] Li J. & Lin H. The current-voltage relation for electropores with conductivity gradients. Biomicrofluidics 4, (2010). 10.1063/1.3324847 PMC2905266 20644669

[b40] Helfrich W. Deformation of lipid bilayer spheres by electric fields. Z. Für Naturforschung Sect. C Biosci. 29, 182–183 (1974). 4276699

[b41] Bryant G. & Wolfe J. Electromechanical stresses produced in the plasma membranes of suspended cells by applied electric fields. J. Membr. Biol. 96, 129–139 (1987). 3599064 10.1007/BF01869239

[b42] Isambert H. Understanding the Electroporation of Cells and Artificial Bilayer Membranes. Phys. Rev. Lett. 80, 3404 (1998).

[b43] Riske K. A. & Dimova R. Electric Pulses Induce Cylindrical Deformations on Giant Vesicles in Salt Solutions. Biophys. J. 91, 1778–1786 (2006). 16766621 10.1529/biophysj.106.081620 PMC1544313

[b44] Popov S. V., Svitkina T. M., Margolis L. B. & Tsong T. Y. Mechanism of cell protrusion formation in electrical field: the role of actin. Biochim. Biophys. Acta BBA - Biomembr. 1066, 151–158 (1991). 10.1016/0005-2736(91)90181-7 1854780

[b45] Needham D. & Hochmuth R. M. Electro-mechanical permeabilization of lipid vesicles. Role of membrane tension and compressibility. Biophys. J. 55, 1001–1009 (1989). 2720075 10.1016/S0006-3495(89)82898-X PMC1330536

[b46] Dimova R. Recent developments in the field of bending rigidity measurements on membranes. Adv. Colloid Interface Sci. 208, 225–234 (2014). 24666592 10.1016/j.cis.2014.03.003

[b47] Goettel M., Eing C., Gusbeth C., Straessner R. & Frey W. Pulsed electric field assisted extraction of intracellular valuables from microalgae. Algal Res. 2, 401–408 (2013).

[b48] Eing C., Goettel M., Straessner R., Gusbeth C. & Frey W. Pulsed Electric Field Treatment of Microalgae -Benefits for Microalgae Biomass Processing. IEEE Trans. Plasma Sci. 41, 2901–2907 (2013).

[b49] Pakhomova O. N. *et al.* Electroporation-induced electrosensitization. PloS One 6, e17100 (2011). 21347394 10.1371/journal.pone.0017100 PMC3036735

[b50] Silve A., Guimerà Brunet A., Al-Sakere B., Ivorra A. & Mir L. M. Comparison of the effects of the repetition rate between microsecond and nanosecond pulses: Electropermeabilization-induced electro-desensitization? Biochim. Biophys. Acta BBA - Gen. Subj. 1840, 2139–2151 (2014). 10.1016/j.bbagen.2014.02.011 24589913

[b51] Silve A., Vézinet R. & Mir L. M. Nanosecond-Duration Electric Pulse Delivery *In Vitro* and *In Vivo*: Experimental Considerations. Instrum. Meas. IEEE Trans. *On* **PP**, 1–10 (2012).

[b52] Silve A., Mir L. M. & Vezinet R. Implementation of a broad band, high level electric field sensor in biological exposure device. in *Power Modulator and High Voltage Conference (IPMHVC), 2010 IEEE International* 711 –714 (2010). doi: 10.1109/IPMHVC.2010.5958458.

